# Obesity is not associated with an increased risk of portal vein thrombosis in cirrhotic patients 

**Published:** 2018

**Authors:** Alan Zakko, Paul T Kroner, Rooma Nankani, Raffi Karagozian

**Affiliations:** 1 *University of Connecticut, School of Medicine, Farmington, CT, United States*; 2 *Department of Gastroenterology and Hepatology, Mayo Clinic, Jacksonville, FL, United States*; 3 *Yale-New Haven Liver Transplantation Center, New Haven, CT, United States *

**Keywords:** Obesity, Portal vein thrombosis, Cirrhosis, ICD-9

## Abstract

**Aim:**

To determine the impact of obesity on development of portal vein thrombosis in cirrhotic patients.

**Background:**

Cirrhosis is a known risk factor for portal vein thrombosis (PVT). Evidence also points to obesity as being a risk factor for venous thromboembolism. Limited information is available on how obesity impacts the development of PVT in cirrhotic patients.

**Methods:**

This was a retrospective cohort study using the 2013 National Inpatient Sample. Patients older than 18 years with an ICD-9 CM code for any diagnosis of liver cirrhosis were included. There was no exclusion criteria. The primary outcome was the impact of obesity on development of PVT. Obesity was also sub-classified according to body-mass index (BMI). Secondary outcomes were in-hospital mortality, ICU admission, shock, TPN use, and resource utilization. Odds ratios (OR) and means were adjusted for age, gender, and ethnicity.

**Results:**

We included 69,934 obese cirrhotics of which, 1,125 developed PVT (mean age 59 years, 35% female). Overall in-hospital mortality rates were 9% (11% with PVT vs 5% without PVT). On multivariate analysis, obesity was not associated with a significantly different adjusted OR for development of PVT compared to non-obese. When stratifying by obesity subtype, class 1 obesity was associated with increased odds of PVT (OR: 1.45, 95%CI: 1.06-1.96, p=0.02), while class 3 obesity was associated with a decreased odds of PVT (OR: 0.72, 95%CI: 0.58-0.88, p<0.01) compared to non-obese.

**Conclusion:**

Obesity is not associated with increased odds of PVT.

## Introduction

 Obesity, defined as a BMI greater than 30 kg/m2, has become a significant health epidemic and is linked to various medical problems (1,2). In recent epidemiological studies, the global prevalence of overweight and obese individuals is estimated to include over one third of the adult population: approximately 36% in men and 38% in women (3). Furthermore, Obesity contributes to an increase in chronic conditions resulting in higher utilization of medical services among broad populations (4). The causal link between obesity and chronic illnesses, such as diabetes and cardiovascular disease, is well established (5). Obesity may also be an independent risk factor for various morbidities including venous thromboembolism (6). It is thought that obesity can interact with environmental or genetic factors and poses a significantly greater risk of venous thromboembolism among higher risk individuals (6). Plausible mechanisms to explain the relation between obesity and venous thrombosis include the existence of a proinflammatory, prothrombotic, and hypofibrinolytic milieu in obese patients (7,8). Emerging evidence suggests that obesity may be an independent risk factor specifically for portal vein thrombosis (PVT) (9).

Portal vein thrombosis (PVT) is not uncommon in patients with advanced cirrhosis and has prevalence between 0.6% and 26% in this population (10). Development of PVT in cirrhotics is associated with various complications (10). Cirrhotics with PVT are at an increased risk of variceal hemorrhage compared to cirrhotics without PVT (10). The development of PVT is also associated with portal biliopathy (biliary duct obstruction due to enlarged collateral veins in contact with the common bile duct) and with intestinal ischemia (10). Moreover, the development of PVT has long been a barrier to successful liver transplantation and is a risk factor for poor outcomes after transplantation (11, 12). 

Despite the known risk of obesity in venous thrombosis, there is limited knowledge regarding the risk of PVT in obese patients. Ayala *et al*. have demonstrated that obesity may be an independent risk factor for pre-transplant PVT in a single center (13). However, no large studies examining the relationship between obesity and the development of PVT in cirrhotic patients have been reported to our knowledge. The aim of this study is to examine if obesity increases the risk of PVT in patients with cirrhosis using a nationwide cohort. 

## Methods

**Design and data source**

The Nationwide Inpatient Sample (NIS), developed by the Healthcare Cost and Utilization Project (HCUP) with the sponsorship of the Agency for Healthcare Research and Quality (AHRQ) that is part of the United States Department of Health and Human Services was used to select all patients included in the study. The NIS database is the largest publicly available inpatient database in the United States. The dataset for the year 2013 contains more than 7.2 million admissions, which in itself are a stratified sample of 20% of hospitalizations, representative of 95% of national hospital discharges from more than 4,300 non-federal acute care hospitals across 44 states in the United States. The dataset includes a principal (or discharge) diagnosis, as well as 24 secondary diagnoses, as well as up to 15 procedural codes for performed procedures during hospitalization. It also includes total length of hospital stay (LOS) and total hospitalization charges. All data for this retrospective cohort study was abstracted from the dataset for the year 2013. 

**Study Population**

International Classification of Diseases, Ninth Revision, Clinical Modification (ICD-9 CM) principal codes were used to identify patients with liver cirrhosis (ICD 9-CM 571.2, 571.5 and 571.6), which were included in the study. Furthermore, the cohort was sub-stratified according to body-mass index (BMI) as non-obese (BMI 18-29kg/m2), obesity class I (BMI 30-34kg/m2), obesity class II (BMI 35-39kg/m2) and obesity class III (BMI≥40 kg/m2) using ICD 9-CM codes. Patients under 18 years of age were excluded from the analysis.

**Variable definition**

The variable information was classified as patient characteristics and hospital characteristics. In terms of patient characteristics, age, gender, race, and median income in zip code were examined. Hospital characteristics included hospital bed-size, teaching status and urban location. Additional variables included patient inpatient mortality, hospital length of stay, total hospitalization charges and the desired outcomes. In considering and accounting for existing patient comorbidities, the Charlson Comorbidity Index was utilized.

**Outcome Measures**

The primary outcome of the study, was the impact of obesity on the development of portal vein thrombosis (PVT). Secondary outcomes included in-hospital mortality, morbidity, measured by shock, intensive care unit (ICU) admission, total parenteral nutrition (TPN) use, and multi-organ failure. Resource utilization was assessed by abdominal computed tomography (CT) and abdominal ultrasound use, as well as hospital length of stay (LOS). Additionally total hospitalization charges and hospital costs were included. 

**Statistical Analysis**

The total number of patients with liver cirrhosis was estimated with the HCUP-published discharge-level weights. Fisher’s exact test and Student’s t test were used to compare proportions and means, respectively. A multivariate logistic regression model was designed by first conducting a literature search of variables relevant to our outcome, which were included in the multivariate regression model if they impacted the outcome in any manner on univariate regression with a p-value of less than 0.1. Using the designed multivariate logistic regression model, odds ratios and means obtained and adjusted for age, gender, race, median income in the patient’s zip code, Charlson Comorbidity Index, hospital region, urban location, hospital bed-size and hospital teaching status. Statistical analysis was conducted with STATA, Version 13 (StataCorp LP, College Station, TX).

## Results

Our cohort included 589,420 patients with liver cirrhosis. This included 69,934 (12%) patients with obesity, of which 1,125 patients had PVT. The mean age of obese cirrhotic patients was 59 years and 35% of them were female. Overall in-hospital mortality rates were 9% (11% with PVT vs 5% without PVT). Obese cirrhotic patients with PVT were similar to non-obese cirrhotic patients with PVT with respect to their age, sex, and race. No statistically significant variation was observed in regards to Charlson Comorbidity Index proportions between these 2 groups (Table 1).

**Table 1:** Patient Characteristics

Patient characteristics	Obese cirrhotics with PVT	Non-obese cirrhotics with PVT	P Value
Age by Category, %			P = 0.15
<25 years old	0.6	0.5	
25-49 years old	15.4	15.4	
>50 years old	84	83.9	
Female, %	34	33	P = 0.0004
Race, %			P = 0.22
White	65.2	65	
Black	9.8	9.9	
Hispanic	16.7	16.9	
Asian or Pacific Islander	3.5	3.7	
Native American	0.9	0.8	
Other	3.9	3.8	
Charlson Comorbidity Index			P = 0.09
Category 0-1	5.9	6	
Category 2-3	3.9	3.7	
Category 3-4	28.2	28.7	
Category ≥ 4	62	61.6	

**Table 2:** Primary and secondary outcomes of obese patients with cirrhosis

Column1	Adjusted OR/mean	95% CI	P-Value
PVT	0.87	0.75-1.0	0.061
PVT Class 1 Obesity	1.45	1.07-1.97	0.017
PVT Class 2 Obesity	0.856	0.53-1.38	0.525
PVT Class 3 Obesity	0.7	0.57-0.86	0.001
Mortality	1.39	0.87-2.23	0.168
Shock	1.04	0.60-1.79	0.884
ICU Admission	1.175	0.754-1.83	0.475
TPN use	1.16	0.42-3.17	0.774
Multiorgan Failure	1.12	0.84-1.49	0.443
Resource Utilization			
CT Abdomen	3.75	1.25-11.35	0.018
US Abdomen	1.57	0.7-3.52	0.274
Length of Stay	0.46	0.68-1.59	0.79

Multivariate regression revealed that obesity did not increase the odds of developing PVT among cirrhotics in our cohort (OR = 0.87, 95% CI: 0.75-1.0, P = 0.061). However, after stratifying patients by obesity subtype, we found increased odds of developing PVT in cirrhotics with class 1 obesity compared to non-obese cirrhotics (OR = 1.45, 95% CI: 1.07-1.97, P = 0.017). In contrast, cirrhotics with class 3 obesity had a decreased odds of developing PVT compared with non-obese cirrhotics (OR = 0.7, 95% CI: 0.57-0.86, P = 0.001). There was no significant difference in the odds for developing PVT in class 2 obesity to non-obese cirrhotics (OR = 0.86, 95% CI: 0.53-1.38, P = 0.525) (Table 2). 

We found no statistically significant differences in the odds for the secondary outcomes of in-hospital mortality (P = 0.168), shock (P = 0.884), ICU admission (P = 0.475), TPN use (P = 0.774), or multiorgan failure (P=0.443) in obese cirrhotics compared to non-obese cirrhotics. While there was no statistically significant differences in the utilization of abdominal ultrasound (P = 0.274), cirrhotics with obesity were at increased odds for utilization of CT scans of the abdomen compared with non-obese cirrhotics (OR = 3.75, 95% CI: 1.25-11.35, P = 0.018).

## Discussion

Portal vein thrombosis is a well-described complication of cirrhosis particularly in the advanced stage of disease. The prevalence of PVT parallels the progression of cirrhosis, being less than 1% in patients with compensated disease, but 8%-25% in liver transplant candidates (14-17). 

Several risk factors for the development of PVT in cirrhotic patients have previously been described. An analysis of adults undergoing liver transplantation in the United Kingdom showed that male sex, previous treatment for portal hypertension, previous splenectomy, Child-Pugh class C, alcoholic liver disease, and hepatocellular carcinoma were all risk factors for PVT (11). Portal system hemostasis and an imbalance of procoagulants in portal hypertension and cirrhosis have also been implicated in the development of PVT (18) (19). Furthermore, Ghabril *et al*. looked at patients in the Organ Procurement and Transplant Network (OPTN) database and suggested that fatty or cryptogenic liver disease, diabetes mellitus, and obesity may be risk factors for development of PVT in patients waiting for liver transplant (12). 

We present the first large scale retrospective cohort analysis to determine the association between obesity and the development of PVT in cirrhosis. Our data suggest that compared to non-obese cirrhotics, there is no increased risk of developing PVT in obese cirrhotic patients. Similar results were found in an observational retrospective study of individuals undergoing liver transplantation showing no differences in the rate of venous thrombotic complications or survival as a function of the BMI class of these liver transplant recipients (20). 

In multivariate subgroup analysis we found increased odds of PVT in cirrhotic patients who also had Type 1 obesity compared with non-obese cirrhotics. This finding further demonstrates that obesity may be associated with increased risk for VTE. In one cohort analysis of the National Hospital Discharge Survey, the risk of developing deep vein thrombosis or pulmonary embolism in obese individuals was significantly increased compared to non-obese (21). Evidence also exists that obese patients have higher plasma concentrations of factors involved in the tissue factor pathway leading to activation of Factor VII and Thrombin (22). Obesity is also associated with platelet activation and several platelet activation markers are found to be increased in obese patients: thromboxane B2, soluble P-selectin, and platelet derived CD40L (22). Furthermore, obesity is associated with increased levels of circulating leptin, a key regulator of body weight and metabolism with known prothrombotic actions. Leptin has been shown to enhance agonist-induced platelet aggregation and increase stability of thrombi (23). The receptor for leptin is expressed on platelets and endothelial cells, and may accelerate thrombus formation by inducing platelet activation, inhibiting vasodilatation, and increasing oxidative stress (24).

Interestingly, we found that class 3 obesity was associated with decreased odds of development of PVT in cirrhosis and the reason for it, is unclear. An observational phenomenon known as the obesity paradox suggests that obesity may confer survival benefit in patients acutely ill (25). In a previous study, Karagozian *et al.* showed that mortality rates among hospitalized patients with cirrhosis are lower in obese patients than in non-obese patients (26). Another potential explanation of lower PVT rates would be that in severe obesity, PVT may be underdiagnosed due to technical difficulties in diagnosis. Sonography has been shown to be limited in obese patients due to increased attenuation as the ultrasound beam passes through larger amounts of subcutaneous and intraperitoneal fat (27). This may also offer an explanation as to why obesity was associated with an almost 4-fold increase in CT scan utilization in our cohort.

Limitations of our study are largely related to the nature of administrative databases. We have to rely on and assume the validity of ICD-9 diagnosis coding for obesity, cirrhosis, and PVT from the NIS administrative data to identify our study population. NIS is also restricted to inpatient admissions; therefore, our data presumes that cirrhotic patients who develop PVT seek hospital admission. We are also unable to determine the exact modality which was used for the diagnosis of PVT and this may have effected odds ratios. There is a potential for not capturing every obesity case due the lack of diagnosis by the clinician; however, this is an inherent limitation of large administrative databases. Patient groups were matched for comorbidities using the Charlson comorbidity index but were not matched specifically for hepatic comorbidities or degree of cirrhosis. Furthermore, a single NIS entry is equivalent to one hospitalization. Thus, a patient may contribute to more than one entry if he or she is readmitted within the study period.

In conclusion, we have found that the overall odds for development of PVT are not significantly different between obese and non-obese cirrhotic patients. When we classified the patients by obesity class 1 and obesity class 3, we found increased and decreased odds Transplantation: Analysis of Risk Factors and Outcomrespectively of development of PVT. Further study is needed to determine what factors may influence a decreased risk of development of PVT in patients with BMI greater than 40.

## Conflict of interests

The authors declare that they have no conflict of interest.
